# Occurrence of testicular microlithiasis in androgen insensitive hypogonadal mice

**DOI:** 10.1186/1477-7827-7-88

**Published:** 2009-08-27

**Authors:** Peter J O'Shaughnessy, Ana Monteiro, Guido Verhoeven, Karl De Gendt, Margaret H Abel

**Affiliations:** 1 Institute of Comparative Medicine, Division of Cell Sciences, University of Glasgow Veterinary School, Bearsden Rd, Glasgow G61 1QH, UK; 2 Laboratory for Experimental Medicine and Endocrinology, Catholic University of Leuven, B-3000 Leuven, Belgium; 3 Department of Human Anatomy and Genetics, University of Oxford, South Parks Rd, Oxford OX1 3QX, UK

## Abstract

**Background:**

Testicular microliths are calcifications found within the seminiferous tubules. In humans, testicular microlithiasis (TM) has an unknown etiology but may be significantly associated with testicular germ cell tumors. Factors inducing microlith development may also, therefore, act as susceptibility factors for malignant testicular conditions. Studies to identify the mechanisms of microlith development have been hampered by the lack of suitable animal models for TM.

**Methods:**

This was an observational study of the testicular phenotype of different mouse models. The mouse models were: cryptorchid mice, mice lacking androgen receptors (ARs) on the Sertoli cells (SCARKO), mice with a ubiquitous loss of androgen ARs (ARKO), hypogonadal (hpg) mice which lack circulating gonadotrophins, and hpg mice crossed with SCARKO (hpg.SCARKO) and ARKO (hpg.ARKO) mice.

**Results:**

Microscopic TM was seen in 94% of hpg.ARKO mice (n = 16) and the mean number of microliths per testis was 81 +/- 54. Occasional small microliths were seen in 36% (n = 11) of hpg testes (mean 2 +/- 0.5 per testis) and 30% (n = 10) of hpg.SCARKO testes (mean 8 +/- 6 per testis). No microliths were seen in cryptorchid, ARKO or SCARKO mice. There was no significant effect of FSH or androgen on TM in hpg.ARKO mice.

**Conclusion:**

We have identified a mouse model of TM and show that lack of endocrine stimulation is a cause of TM. Importantly, this model will provide a means with which to identify the mechanisms of TM development and the underlying changes in protein and gene expression.

## Background

Testicular microlithiasis (TM) is characterised by the presence of microcalcification within the seminiferous tubules. In the normal human population the incidence is between 1.5 and 5.6% [1-3] and in itself microlithiasis is benign but there may be significant association with malignant conditions such as testicular germ cell tumors (TGCT) [4-7] and other conditions such as cryptorchidism, varicocele, infertility, and testicular torsion [8,9]. In addition, it has been suggested that there may be a genetic predisposition to TM which is linked to TGCT formation so that identifying the underlying causes of microlithiasis may help identify susceptibility factors for TGCTs [5]. Development of an animal model of TM would, therefore, not only represent significant progress towards an understanding of the origins and underlying molecular mechanisms of microlithiasis but may help identify associated risk factors for other conditions.

The hypogonadal (*hpg*) mouse lacks gonadotrophin-releasing hormone (GnRH) and circulating levels of luteinising hormone (LH) and follicle-stimulating hormone (FSH) are undetectable [10]. As part of an ongoing study of the endocrine regulation of spermatogenesis we have crossed these *hpg *mice with mice lacking androgen receptors (AR) either ubiquitously (ARKO mice) or specifically in the Sertoli cells (SCARKO mice). In this report we describe the unexpected and consistent occurrence of microlithiasis in *hpg*.ARKO mice.

## Methods

### Animals and treatments

All mice were bred and all procedures carried out under UK Home Office Licence and with the approval of a local ethical review committee. SCARKO and ARKO mice have been previously generated by crossing female mice carrying an *Ar *with a floxed exon 2 (*Ar*^*fl*^) with male mice expressing Cre under the regulation of the Sertoli cell specific promoter of AMH or the ubiquitous promoter PGK-1 [11,12]. In order to produce *hpg*.SCARKO mice, *hpg *mice (C3HE/HeH-101/H) were initially crossed with mice carrying the *Ar*^*fl *^allele (Swiss-Webster/129) and with mice expressing AMH-Cre (C57-BL6/SJL). From these crosses, female mice heterozygous for the GnRH deletion (*hpg*/+) and homozygous for the *Ar*^*fl *^allele were crossed with *hpg*/+ AMH-Cre males (heterozygous or homozygous for the Cre transgene) to generate *hpg*.SCARKO mice. The *hpg *deletion and *Ar*^*fl *^allele were detected by PCR analysis of ear lysates and confirmed at termination by the presence of the shorter *Ar *allele in a testis extract [12,13]. The generation of *hpg*.ARKO mice was similar except that PGK-Cre (C57-BL6/SJL) replaced AMH-Cre. The *hpg*.ARKO males were detected by PCR of ear lysates for *Sry *and deletion of GnRH and confirmed at termination by the absence of epididymides, seminal vesicles and ductus deferens. Cryptorchidism was induced in normal 20-day old mice (C3H/HeH-101/H) as described previously [14].

To determine the effects of hormone treatment, adult *hpg*, *hpg*.SCARKO and *hpg*.ARKO mice (10 weeks of age) were injected sub-cutaneously with 8 IU recombinant human FSH (rhFSH) (Serono Ltd, London, UK) in 0.2 ml PBS (phosphate buffered saline, pH 7.4, Sigma Aldrich, Poole, UK) once daily or had silastic tubing (2 cm) containing either testosterone (T) or dihydrotestosterone (DHT) implanted subcutaneously. Treatments lasted for 7 days.

Adult mice were euthanized at 9-11 weeks of age by cervical dislocation and the testes were fixed overnight in Bouin's solution.

### Histology and microlith numbers

Testes were embedded in Technovit 7100 resin, cut into sections (20 μm), and stained with Harris' hematoxylin. Microliths were counted in every 6^th ^section to generate an estimate of numbers per testis.

### Statistical analysis

Data were analysed using single factor analysis of variance followed by Fisher's test for multiple comparisons. All data are expressed as mean ± SEM.

## Results

**Figure 1:**
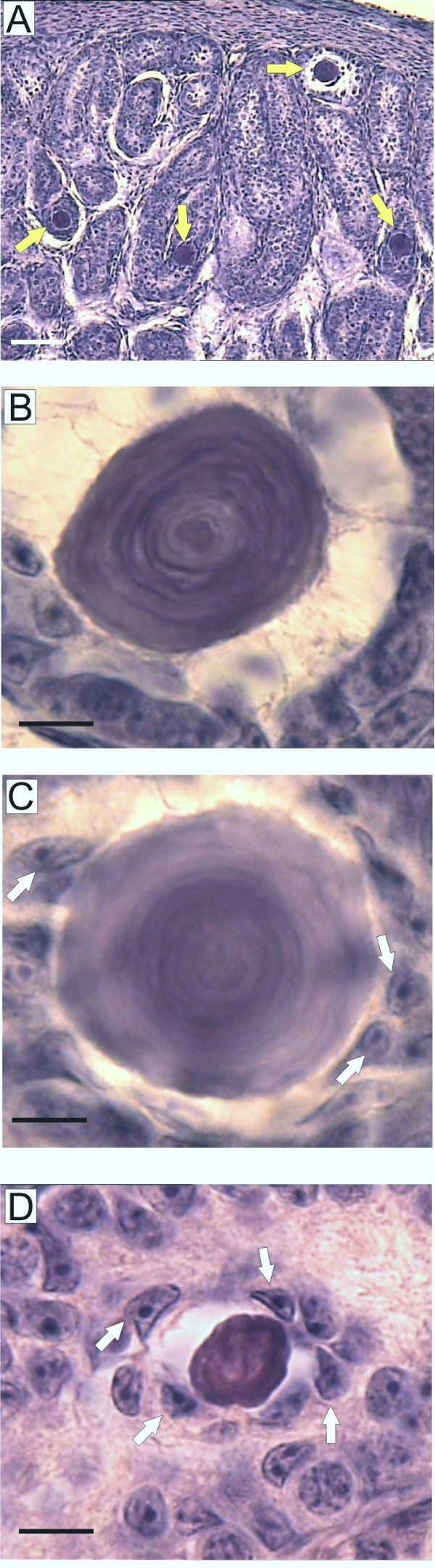
**Microliths in *hpg *and *hpg*.ARKO mice**. Tissue sections showing microliths in *hpg*.ARKO (A, B, C) and *hpg *(D) mice. In A) the microliths are identified by yellow arrows. In C) and D) the white arrows identify Sertoli cells apposed to the microliths. The line represents 100 μm in A) and 10 μm in B)-D).

Microscopic analysis of *hpg*.ARKO mice showed that in 94% (n = 16) of these animals non-cellular structures, closely resembling the morphology of human testicular microliths [15], were present within the seminiferous tubules (Fig 1). In section the microliths had a round/oval appearance with a maximum diameter of about 45 μm (Fig 1). Most microliths were present singly but in some sections 2 or 3 microliths were present within the same tubule section. Some microliths were closely apposed to Sertoli cells while others had only occasional close cellular associations (Fig 1B&1C).

Examination of *hpg *and *hpg*.SCARKO mice showed that occasional, small microliths were present in 36% (n = 11) of *hpg *testes and 30% (n = 10) of *hpg*.SCARKO testes (Fig 1D). We did not observe any evidence of the presence of microliths in ARKO mice (n = 4) or in SCARKO mice (n = 7) or in normal cryptorchid mice (n = 4).

The mean number of microliths/testis in *hpg*.ARKO mice was 81 ± 54 while in *hpg *and *hpg*.SCARKO mice there were 2 ± 0.5 and 8 ± 6 microliths per testis respectively. Treatment of *hpg*, *hpg*.SCARKO or *hpg*.ARKO mice with FSH did not alter the number of microliths. Treatment of mice with androgen increased the average number of microliths per testis in *hpg*.ARKO mice but this was not significant (incidence after T = 234 ± 75, incidence after DHT = 241 ± 75).

## Discussion

The generation of an animal model of TM represents significant progress towards understanding this condition. It is unlikely that the cause of TM in the *hpg*.ARKO mouse is identical to that in most human cases of TM since TM arises in individuals with normal endocrinology although, interestingly, TM has been shown to be diagnostic of McCune-Albright Syndrome [16] in which testicular symptoms appear to arise most commonly from precocious activation of the Sertoli cells [17]. Irrespective of the cause, importantly we have identified conditions in which there is consistent development of TM and the molecular mechanisms involved in generation of TM in *hpg*.ARKO mice are likely to be similar to those in the human. This mouse model can be used, therefore, to study the generation and characteristics of TM including identification of changes in gene expression and protein secretion associated with formation of TM. In addition, the *hpg*.ARKO mouse now provides a chance to trace the origins and physical development of testicular microliths.

In humans TM is normally diagnosed ultrasonically by the presence of multiple 1-3 mm echogenic foci which are clearly far larger than the structures observed microscopically in this study. This is likely to be a reflection of the differences in testis size and tubule diameter between human and *hpg*.ARKO mouse testes and the morphology of the microliths appears very similar between mouse and human [15]. Nevertheless, it should be noted that the criteria used to define TM in this study is microscopic, rather than ultrasonic, and direct extrapolation to the human condition must be made with caution. Clearly, in this respect, it will be of interest to determine whether there is any development of TGCT in *hpg*.ARKO mice as they age beyond early adulthood.

The origin of testicular microliths is uncertain. Most studies favour a tubular origin with accumulation of debris from degenerating cells being followed by secretion of glycoproteins and subsequent calcification [18,19]. Others suggest that microliths originate outside the tubule and then either move into the tubule or become engulfed by the tubule folding around it [15,20]. Developmental studies of TM in the *hpg*.ARKO mouse should provide a clear answer to the origin of microliths. It may be of interest, however, that microliths are much more prevalent in *hpg*.ARKO mice than in *hpg*.SCARKO mice since one of the differences between these two animal models lies in the presence of ARs in the extratubular space.

It is unlikely that microliths form consistently in the *hpg*.ARKO mouse simply because there is abundant degeneration of the germ cells. This may be a contributory factor but there is marked degeneration of germ cells in *hpg*, SCARKO and ARKO mice and only in *hpg *mice did we see any evidence of occasional microliths in some animals. Similarly, cryptorchidism may be a contributory factor to microlith formation in *hpg*.ARKO mice but is unlikely to be the principal cause as *hpg *and ARKO mice are cryptorchid and no evidence of microliths was seen in normal animals rendered cryptorchid by surgical means. In *hpg*.ARKO mice the epididymides fail to form which means that there will be a block to the flow of fluid through the seminiferous tubules. This may also be a contributory factor to the formation of microliths in the *hpg*.ARKO mouse but is unlikely to be the major cause since there is a similar failure of epididymal development in ARKO mice. Removal of gonadotrophin stimulation is clearly a contributory factor to microlith formation as, in itself, it appears to be sufficient to induce formation of occasional microliths in the *hpg *mouse. Overall, therefore, it may be a combination of factors (degenerating cells, cryptorchidism, lack of tubular fluid flow), linked to loss of gonadotrophin- and androgen-stimulation, that leads to consistent TM development in the *hpg*.ARKO mouse.

## Conclusion

We have shown that the *hpg*.ARKO mouse consistently develops TM. This provides a new and valuable model for studying the cellular and molecular mechanisms involved in TM development.

## Abbreviations

AR: androgen receptor; ARKO: androgen receptor knockout; DHT: dihydrotestosterone; FSH: follicle-stimulating hormone; Gnrh: gonadotrophin-releasing hormone; hpg: hypogonadal; SCARKO: Sertoli cell androgen receptor knockout; T: testosterone; TGCT: testicular germ cell cancer; TM: testicular microlithiasis

## Competing interests

The authors declare that they have no competing interests.

## Authors' contributions

POS identified the presence of microliths within the testes, drafted the manuscript and obtained funding. AM carried out the histological analysis. GV and KdeG generated animal models and revised the manuscript. MHA generated animal models, carried out hormone treatments, obtained funding and revised the manuscript. All authors have read and approved the final manuscript.
